# Circular RNA pappalysin-1 enhances glycolysis via microRNA-656-3p targeting G-protein subunit gamma-5 to promote colon cancer progression

**DOI:** 10.1016/j.clinsp.2025.100594

**Published:** 2025-02-13

**Authors:** AiYuan Cai, HuiShi Ye, YuanHong Lin, JinYun Li, DongSheng Fang, ZhongBin Pan, ZhiWei Li, GuangLiang Luo, YanFang Huang, CiAi Lai

**Affiliations:** a Department of Paediatrics, Shenzhen Hospital (Futian) of Guangzhou University of Chinese Medicine, Shenzhen City 518034, Guangdong Province, PR China; b Department of Paediatrics, Dongguan Hospital of Guangzhou University of Chinese Medicine, Dongguan City, Guangdong Province, PR China; c Second Clinical Medical College, Guangzhou University of Chinese Medicine, Guangzhou City, Guangdong Province, PR China; d Acupuncture Rehabilitation Clinical College, Guangzhou University of Chinese Medicine, Guangzhou City, Guangdong Province, PR China; e Xi 'an Jiaotong University, Xi 'an City, Shaanxi Province, PR China

**Keywords:** Colon cancer, circ-PAPPA, GNG5, Proliferation, Glycolysis

## Abstract

**Background and objective:**

Colon Cancer (CC) is a common malignant tumor. The aim of this study was to investigate the role and regulatory mechanism of circular RNA pappalysin-1 (circ-PAPPA; hsa_circ_0088233) in CC.

**Methods:**

In cancer tissues from CC patients, circ-PAPPA expression was measured and its relationship with patients’ clinical features was analyzed. Plasmid vectors or oligonucleotides interfering with the expression of circ-PAPPA, microRNA (miR)-656–3p or G-protein subunit Gamma-5 (GNG5) were transfected into CC cells. Cell viability was detected by MTT and colony formation assay; apoptosis was detected by flow cytometry; and cell migration and invasion were detected by wound healing assay and Transwell. Glycolytic capacity of CC cells was assessed by measuring glucose uptake and lactate production using commercial kits. The targeting relationship between miR-656–3p and circ-PAPPA or GNG5 was verified by bioinformatics website starBase and dual luciferase reporter gene assay assays.

**Results:**

Circ-PAPPA was upregulated in CC and was negatively correlated with benign pathological features and 5-year survival rates of CC patients. Circ-PAPPA silencing inhibited the growth and glycolysis of CC cells through upregulating miR-656–3p. GNG5, a target of miR-656–3p, could reverse the impacts of silencing circ-PAPPA on CC cells.

**Conclusion:**

Circ-PAPPA may play an oncogenic role in CC by promoting cell growth and glycolysis through the miR-656–3p/GNG5 axis.

## Introduction

Colon Cancer (CC), a malignant tumor of the digestive system, is frequently associated with high rates of illness and death.^1^ Its occurrence is steadily rising, largely due to unhealthy lifestyle choices.^2^ Currently, radiotherapy and chemotherapy are the first-line treatments for resectable and advanced CC.^3^ However, advanced CC has extremely poor 5-year survival rates due to a lack of effective diagnostic biomarkers and inconspicuous early symptoms.^4^ In addition, the risk of recurrence is extremely high due to distant metastasis.^5^ In light of this, new early diagnosis and treatment strategies are urgently needed.

It is currently accepted that aerobic glycolysis plays a crucial role in cancer progression.^6^ Different from normal cells, cancer cells mainly acquire the ability to survive and reproduce through aerobic glycolysis (Warburg effect).^7^ Abnormal cancer cell metabolism is a hallmark of cancer, among which aerobic glycolysis (oxidation of glucose to pyruvate and then lactate under normoxia) early indicates altered tumor metabolism.^8^ Therefore, suppression of glycolysis holds great therapeutic promise in tumor therapy. For example, loss of Alpha/beta hydrolase domain-containing protein 5 promotes colorectal cancer development by inducing aerobic glycolysis^9^ and Ezrin induces CC cell proliferation and migration via glycolysis.^10^

Circular RNAs (circRNAs) are single-stranded closed RNAs that are more stable than linear RNAs. CircRNAs play key roles in gene regulation, including the formation of microRNAs (miRNAs) and the regulation of protein translation.^11^, ^12^, ^13^ Recently, more and more studies have shown that circRNAs can regulate aerobic glycolysis in cancers. For example, circCDKN2B-AS1 promotes aerobic glycolysis by adsorbing insulin-like growth factor-2 mRNA binding protein-3 to stabilize hexokinase-2 mRNA, thereby promoting the malignant progression of cervical cancer.^14^ By enhancing aerobic glycolysis, circ-PDCD11 promotes triple-negative breast cancer development.^15^ Circular RNA pappalysin-1 (circ-PAPPA) is a circRNA discovered to be up-regulated in prostate cancer and it can promote prostate cancer progression.^16^ However, its expression and regulatory mechanisms in CC remain unclear.

This study aims to explore the role of circ-PAPPA in CC and its underlying mechanism. It is hypothesized that circ-PAPPA can enhance aerobic glycolysis and promote CC progression. To confirm the hypothesis, the expression of circ-PAPPA in CC was first determined, and then the effects of circ-PAPPA on CC cell viability, apoptosis, migration, invasion, and aerobic glycolysis were analyzed.

## Materials and methods

### Clinical samples

The clinical part of this study followed the STROBE guidelines. Forty pairs of tumor and non-tumor specimens (5 cm from the tumor legion) were obtained from CC patients (age range: 42‒78-years; mean age, 57-years) who underwent surgical resection in Shenzhen Hospital of Guangzhou University of Chinese Medicine. None of these patients received preoperative anticancer therapy. All procedures were approved by the Ethics Committee of Shenzhen Hospital of Guangzhou University of Chinese Medicine (Approval nº 2017GZ60931). Each subject participating in this study provided written informed consent.

### Cell culture

Human normal colorectal mucosal cells (FHC) and CC cell lines LoVo, HT-29, HCT116, and SW480 (BeNa Culture Collection; Beijing, China) were placed in a culture system consisting of Dulbecco's Modified Eagle's Medium (DMEM; Gibco, Thermo Fisher Scientific, MA, USA), 10 % fetal bovine serum (FBS; Hyclone; GE Healthcare Life Sciences, UT, USA) and 100 U/mL penicillin/streptomycin (Gibco; Thermo Fisher Scientific). The medium was renewed every 2 or 3 days.^17^

### Cell transfection

miR-656–3p mimic (5′-AAUAUUAUACAGUCAACCUCU-3′), mimic NC (5′-GGUAGAUAGGAUUGUUAGGCGAC-3′), miR-656–3p inhibitor (5′-AGAGGUUGACUGUAUAAUAUU-3′), and inhibitor NC (5′-CAGUACUUUUGUGUAGUACAA-3′) were purchased from GenePharma (Shanghai, China). Small interfering RNA targeting circ-PAPPA (si-PAPPA) and plasmid oe-circ-PAPPA (oe-PAPPA), sh-GNG5, along with their negative controls were obtained (Invitrogen, CA, USA). Lipofectamine 3000 (Invitrogen; Thermo Fisher Scientific) was used to transfect plasmids or oligonucleotides into cells. .^16^

### Detection of viability

An analysis of cell viability was performed using 3-(4, 5-dimethylthiazol-2-yl)−2, 5-diphenyltetrazolium bromide assay kit and measurements of optical density_570__nm_ were done using a 96-well plate reader with a reference wavelength of 630 nm.^18^

### Evaluation of colony-forming ability

Approximately 500 cells were cultured for 14 days and stained with 10 % Giemsa solution (Merck, Germany). Colonies containing 50 cells were counted under a microscope (Olympus, Tokyo, Japan).^19^

### Apoptosis assay

Cells after detachment were stained with propidium iodide and annexin V-fluorescein isothiocyanate. Cell apoptosis was analyzed on a flow cytometer (BD Biosciences, CA, USA).^20^

### Wound healing test

Cell wounds were made by a 10-μL pipette tip when cells were completely confluent. Wounds were then recovered by adding FBS-free DMEM (HyClone) in the cell culture medium. After 24 h, the area occupied by migrating cells was measured under a microscope (Olympus) (Cell migration rate = area occupied by migrated cells/original area × 100 %).^21^

### Invasion assay

The upper chamber of Transwell (Costar, Corning, USA) was precoated with 40 μL of Matrigel (1:8; BD Biosciences), in which 5 × 10^4^ cells cultured in 100 μL of serum-free medium were added. Cells were chemo-attracted by 500 μL of 10 % FBS medium in the lower chamber. The invading cells after 24 h were fixed with 4 % paraformaldehyde (Sigma-Aldrich; Merck KGaA), stained with 0.5 % crystal violet (Sigma-Aldrich; Merck KGaA), and counted in 5 random fields.^22^

### Glucose uptake and lactate production assay

Glucose Uptake Colorimetric Assay Kit (Biovision, CA, USA) was used to detect glucose uptake. Lactate Assay Kit (Sigma, MO, USA) was applied for the detection of lactate production.^23^

### RNA determination

Total RNA was extracted using Trizol (TransGen Biotech, Beijing, China) and analyzed by NanoDrop-1000 (Thermo Fisher, Waltham, MA, USA) to check the concentration. Reverse transcription to cDNA was implemented using a High-Capacity cDNA kit (Thermo Fisher) or MiX-x miRNA synthesis kit (TaKaRa, Dalian, China). Circ-PAPPA, miR-656–3p, and GNG5 levels were quantified by SuperReal PreMix Color mix (Tiangen, Beijing, China) and evaluated by the 2^−ΔΔCt^ method, taking U6 and Glyceraldehyde 3-Phosphate Dehydrogenase (GAPDH) as internal controls, respectively. Table 1 lists the sequences of primers.^24^

**Table 1:** Gene sequences for PCR.

Genes	Sequences (5′–3′)
circ-PAPPA	F: ATACCATGTGCCTGGATCCTC
	R: GCCATCACCATTGATCTTATT
miR-656–3p	F: CGCGCAATATTATACAGTCA
	R: GCAGGGTCCGAGGTATTC
GNG5	F: AGCACAGAACCGGAAACTTAG
	R: TCACTTTTACGCGGTTGAGTC
U6	F: CTCGCTTCGGCAGCACA
	R: AACGCTTCACGAATTTGCGT
GAPDH	F: CACCCACTCCTCCACCTTTG
	R: CCACCACCCTGTTGCTGTAG

F, Forward; R, Reverse; circ-PAPPA, Circular RNA Pappalysin-1; miR-656–3p, microRNA-656–3p; GNG5, G-protein subunit Gamma-5; GAPDH, Glyceraldehyde 3-Phosphate Dehydrogenase.

### Protein detection

Total protein was extracted using radioimmunoprecipitation assay lysis buffer (Invitrogen; Thermo Fisher Scientific), followed by an examination of protein concentration using the BCA kit (Beyotime, Haimen, China). The quantified protein was then denatured by sodium dodecyl sulfate-polyacrylamide gel electrophoresis and transferred to a polyvinylidene fluoride membrane (Amersham Biosciences, USA). Then, the membrane blocked with 5 % nonfat milk was counteracted with primary antibodies GNG5 (ab238835, 1:1000) and GAPDH (ab8245, 1:1000; Abcam) as well as horseradish peroxidase-labeled secondary antibody (1:5000; Abcam) and viewed under an optical luminometer (GE, TX, USA).^25^

### Dual-luciferase reporter assay

On the starBase (http://starBase.sysu.edu.cn/), candidate downstream targets of circ-PAPPA and miR-656–3p were predicted, respectively. The vectors (PAPPA-WT, PAPPA-MUT, GNG5-WT, and GNG5-MUT) were produced by inserting circ-PAPPA or GNG5 3′UTR wild-type or mutant sequences containing miR-656–3p putative binding site into psiCHECK2.0 vector (Promega, WI, USA). SW480 cells seeded at 4 × 10^4^ cells/well were co-transfected with the vector and mimicked NC or miR-656–3p mimic using Lipofectamine 3000, and assayed for luciferase activity using Dual-Luciferase Assay System Kit (Promega).^26^

### Statistical analysis

All data were processed using SPSS 21.0 (IBM, NY, USA), measurement data were expressed as mean ± standard deviation. Data in normal distribution were compared by *t*-test between two groups. Comparisons among multiple groups were done with a one-way analysis of variance and Tukey's post hoc test. The repeated-measures analysis of variance was used to compare data between groups at different time points, followed by a Bonferroni post hoc test. Pearson correlation analysis was done in clinical samples; p-value < 0.05 was indicative of a significant difference.

## Results

### Circ-PAPPA is overexpressed in CC

**Fig. 1:**
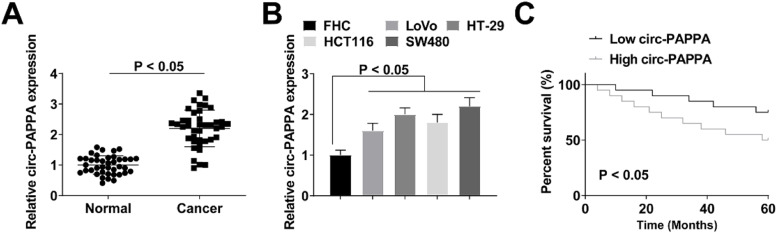
**circ-PAPPA is overexpressed in CC.** circ-PAPPA expression in clinical samples and cells (A‒B); Correlation analysis of circ-PAPPA expression with overall survival of CC patients (C); values were expressed as mean ± standard deviation.

circ-PAPPA expression was significantly upregulated in CC tissues (Fig. 1A). Increased expression of circ-PAPPA was shown in CC cell lines, among which SW480 cells exhibited the most significant changes in circ-PAPPA expression (Fig. 1B), so it was selected for subsequent experiments. Regarding the correlation between circ-PAPPA expression and clinical characteristics of CC patients, 40 patients were divided into two groups, with the median expression level of circ-PAPPA as the cutoff point. It was determined that circ-PAPPA expression was associated with TNM stage, lymphatic metastasis, tumor size, and distant metastasis (Table 2); and circ-PAPPA expression had a negative correlation with patients’ survival (Fig. 1C).

**Table 2:** Demographic and clinicopathological characteristics of colon cancer patients.

Characteristics	Case	circ-PAPPA expression		p-value
		Low (*n* = 20)	High (*n* = 20)	
Age (years)				0.527
≤ 60	21	12	9	
> 60	19	8	11	
Gender				0.751
Male	22	10	12	
Female	18	10	8	
Tumor node metastasis stage				0.010
*I* + II	17	13	4	
III + IV	23	7	16	
Lymphatic metastasis				<0.001
N0/N1	20	16	4	
N2/N3	20	4	16	
Tumor size (cm)				0.023
≤ 3	16	12	4	
> 3	24	8	16	
Distant metastasis				0.008
Negative	25	17	8	
Positive	15	3	12	

### Downregulating circ-PAPPA inhibits CC growth and glycolysis

**Fig. 2:**
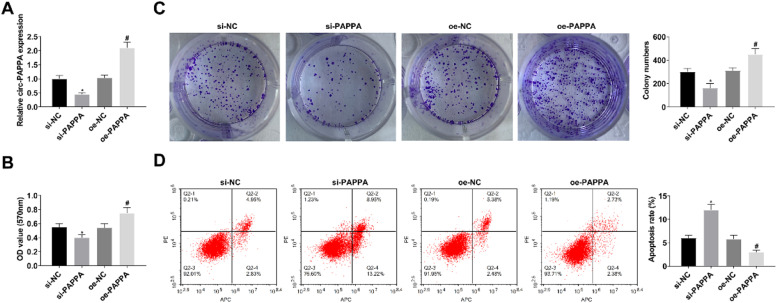
**Downregulating circ-PAPPA inhibits CC cell proliferation and promotes apoptosis.** RT-qPCR to verify the intervention of circ-PAPPA expression (A); MTT and colony formation assays to detect cell proliferation (B‒C); flow cytometry to detect cell apoptosis (D); values were expressed as mean ± standard deviation (*n* = 3). * p-value < 0.05 vs. si-NC; # p-value < 0.05 vs. oe-NC.

**Fig. 3:**
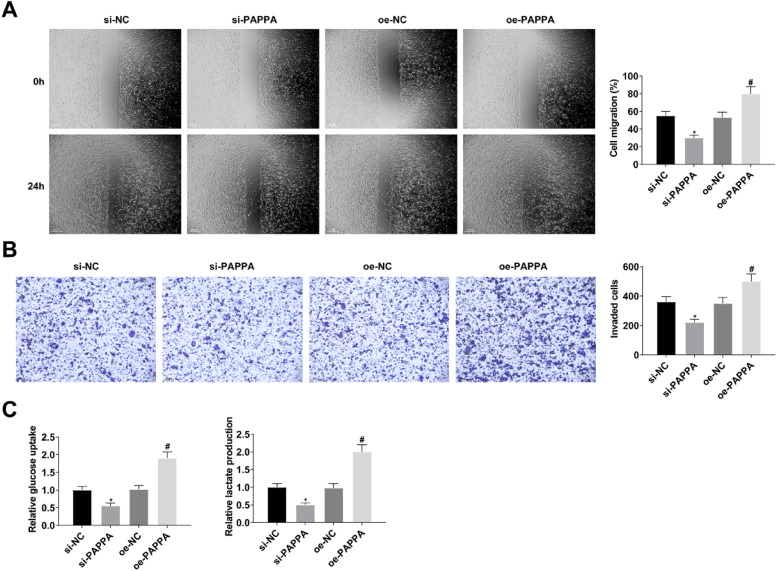
**Downregulating circ-PAPPA inhibits CC cell migration, invasion and glycolysis.** Scratch assay and Transwell assay for cell migration and invasion (A‒B); Glucose uptake colorimetric kit and lactate content colorimetric kit for cellular glucose uptake and lactate production levels (C); values were expressed as mean ± standard deviation (*n* = 3). * p-value < 0.05 vs. si-NC; # p-value < 0.05 vs. oe-NC.

When studying the effect of circ-PAPPA on CC, si-PAPPA, si-NC, oe-PAPPA, and oe-NC were transfected into SW480 cells, respectively, and the successful transfection was verified (Fig. 2A). Then, cell proliferation was detected by MTT and colony formation assay, and the results showed that down-regulation of circ-PAPPA inhibited cell proliferation and up-regulation of circ-PAPPA promoted cell proliferation (Fig. 2B‒C); apoptosis was detected by flow cytometry, and the results showed that down-regulation of circ-PAPPA promoted apoptosis and up-regulation of circ-PAPPA inhibited cell apoptosis (Fig. 2D); cell migration and invasion were detected by scratch assay and Transwell, and the results showed that down-regulation of circ-PAPPA inhibited cell migration and invasion, and up-regulation of circ-PAPPA promoted cell migration and invasion (Fig. 3A‒B); cellular glucose uptake and lactate production levels were detected by glucose uptake colorimetric kit and lactate colorimetric kit, and the results showed that down-regulation of circ-PAPPA inhibited cellular glucose uptake and lactate production, and up-regulation of circ-PAPPA promoted cellular glucose uptake and lactate production (Fig. 3D). These results suggest that down-regulation of circ-PAPPA inhibits CC growth and glycolysis.

### Circ-PAPPA inhibits miR-656–3p expression

**Fig. 4:**
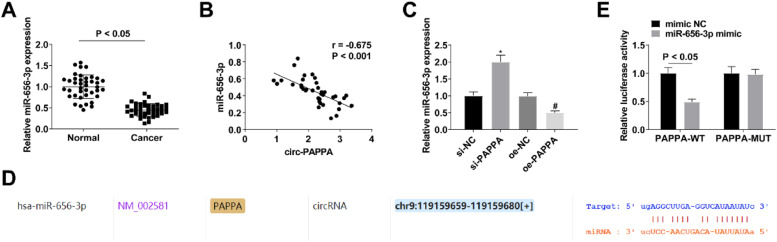
**Circ-PAPPA inhibits miR-656–3p expression.** miR-656–3p expression levels in clinical samples (A), correlation analysis of circ-PAPPA and miR-656–3p expression levels (B), miR-656–3p expression after intervening circ-PAPPA (C), the binding site of circ-PAPPA and miR-656–3p on starBase database (D) and cell luciferase activity (E); values were expressed as mean ± standard deviation (*n* = 3). *p-value < 0.05 vs. si-NC; #p-value < 0.05 vs. oe-NC.

miR-656–3p was poorly expressed in CC tissues (Fig. 4A) and was negatively related to circ-PAPPA expression (Fig. 4B). Subsequently, whether circ-PAPPA could alter the expression of miR-656–3p was explored in SW480 cells, and it was found that the expression of miR-656–3p changed inversely with the changes in circ-PAPPA expression (Fig. 4C). Next, starBase database predicted that circ-PAPPA has a binding site with miR-656–3p (Fig. 4D). This binding relationship was further confirmed by reduced luciferase activity in SW480 cells by co-transfection of PAPPA-WT and miR-656–3p mimic (Fig. 4E).

### Upregulating miR-656–3p suppresses CC growth and glycolysis

**Fig. 5:**
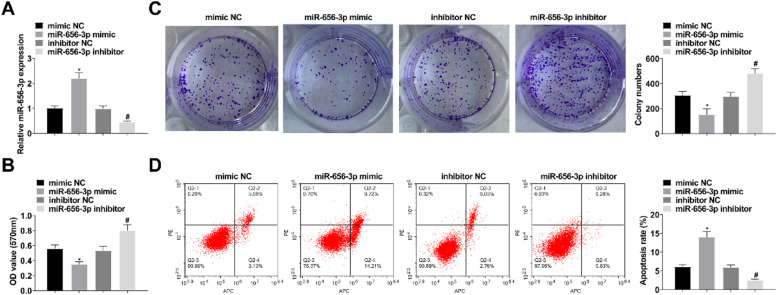
**Upregulating miR-656–3p suppresses CC cell proliferation and promotes apoptosis.** RT-qPCR to verify the intervention of miR-656–3p expression (A); MTT and colony formation assays to detect cell proliferation (B‒C); flow cytometry to detect cell apoptosis (D); values were expressed as mean ± standard deviation (*n* = 3). * p-value < 0.05 vs. mimic NC; # p-value < 0.05 vs. inhibitor NC.

**Fig. 6:**
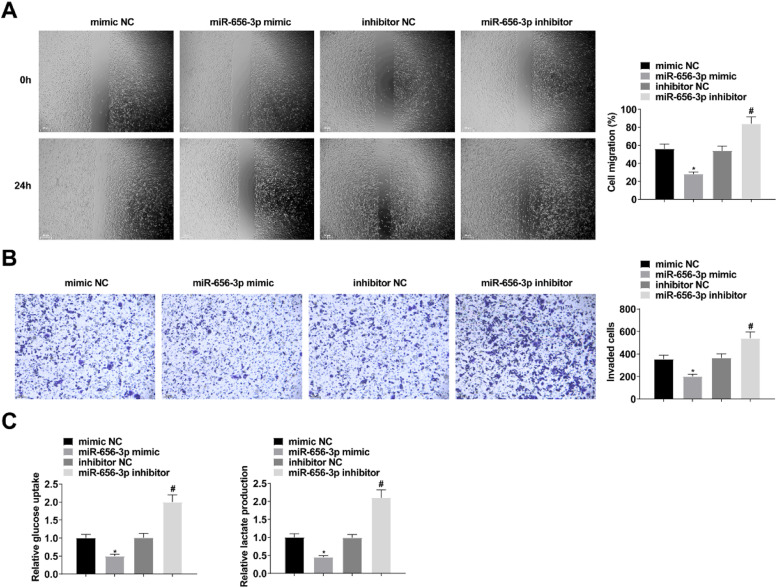
**Upregulating miR-656–3p depresses CC migration, invasion and glycolysis.** Scratch assay and Transwell assay for cell migration and invasion (A‒B); Glucose uptake colorimetric kit and lactate content colorimetric kit for cellular glucose uptake and lactate production levels (C); values were expressed as mean ± standard deviation (*n* = 3). * p-value < 0.05 vs. mimic NC; # p-value < 0.05 vs. inhibitor NC.

miR-656–3p expression was altered by transfection of miR-656–3p mimic/inhibitor in SW480 cells (Fig. 5A). The above experiments revealed that up-regulation of miR-656–3p inhibited the growth, glucose uptake and lactate production of SW480 cells, whereas down-regulation of miR-656–3p promoted the growth, glucose uptake and lactate production of SW480 cells (Fig. 5B‒D; Fig. 6A‒C).

### miR-656–3p could decorate GNG5 expression

**Fig. 7:**
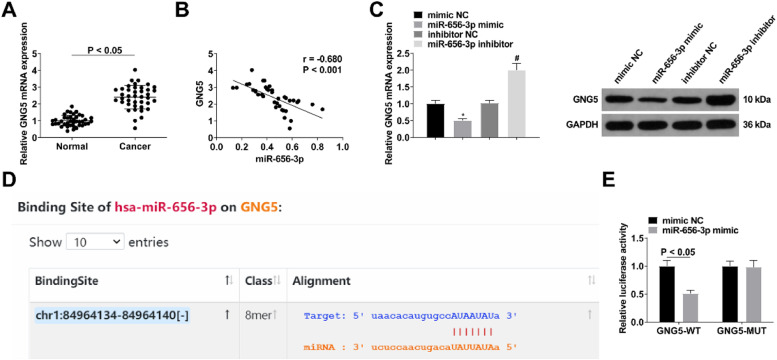
**miR-656–3p could decorate GNG5 expression.** GNG5 expression levels in clinical samples (A), correlation analysis of GNG5 and miR-656–3p expression levels (B), GNG5 expression after intervening miR-656–3p (C), the binding site of GNG5 and miR-656–3p on starBase database (D) and cell luciferase activity (E); values were expressed as mean ± standard deviation (*n* = 3). *p-value < 0.05 vs. mimic NC group; # p-value < 0.05 vs. inhibitor NC.

An increase in GNG5 expression was determined in clinical CC samples (Fig. 7A). Based on this, a targeting relationship between miR-656–3p and GNG5 was hypothesized. Pearson analysis revealed the inverse correlation between miR-656–3p and GNG5 expression in CC tissues (Fig. 7B). Up- and down-regulation of miR-656–3p reduced and promoted GNG5 expression, respectively (Fig. 7C). Afterwards, starBase found the binding sites of miR-656–3p to GNG5 (Fig. 7D) and this targeting relationship was confirmed by reduced luciferase activity in SW480 cells transfected with miR-656–3p mimic and GNG5-WT (Fig. 7E).

### Knockdown of GNG5 reverses the effect of upregulation of circ-PAPPA on CC

**Fig. 8:**
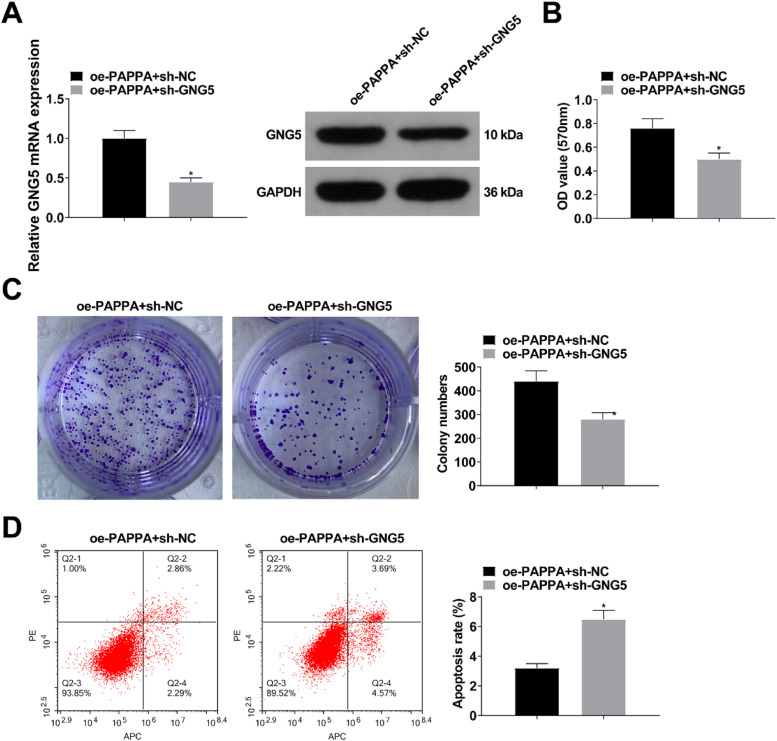
**Knockdown of GNG5 reverses the effect of upregulation of circ-PAPPA in CC.** RT-qPCR and Western blot to verify the intervention of GNG5 expression (A); MTT and colony formation assays to detect cell proliferation (B‒C); flow cytometry to detect cell apoptosis (D); values were expressed as mean ± standard deviation (*n* = 3). *p-value < 0.05 vs. oe-PAPPA + sh-NC.

**Fig. 9:**
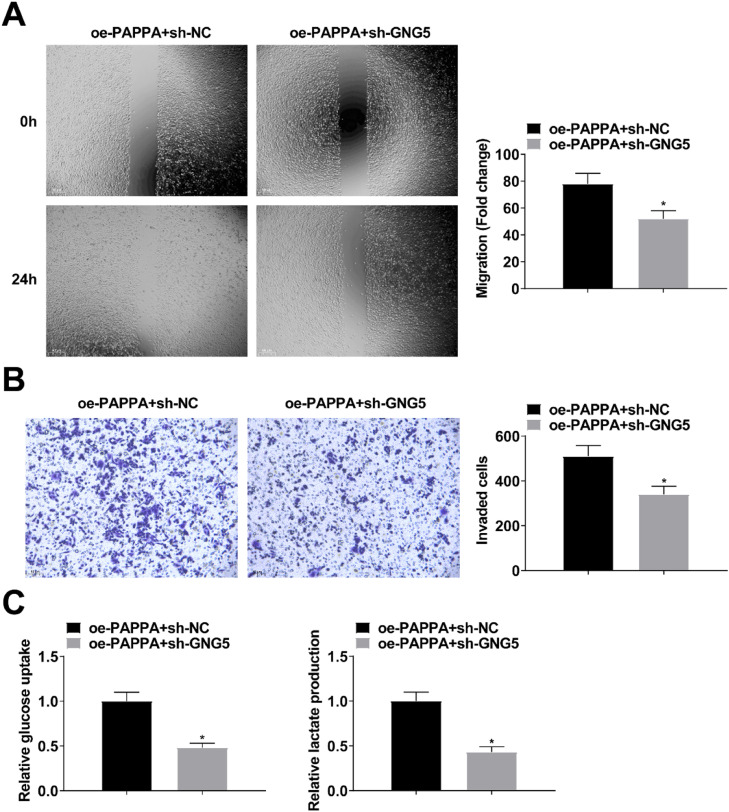
**Knockdown of GNG5 reverses the effect of upregulation of circ-PAPPA in CC.** Scratch assay and Transwell assay for cell migration and invasion (A‒B); Glucose uptake colorimetric kit and lactate content colorimetric kit for cellular glucose uptake and lactate production levels (C); values were expressed as mean ± standard deviation (*n* = 3). * p-value < 0.05 vs. oe-PAPPA + sh-NC.

The involvement of GNG5 in circ-PAPPA affecting CC cell activity was investigated by transfecting PAPPA-expressing SW480 cells with sh-GNG5. RT-qPCR and Western blot results showed that down-regulation of GNG5 attenuated the promotion of GNG5 expression by up-regulation of PAPPA (Fig. 8A). PAPPA overexpression-mediated SW480 cell growth and glycolysis were suppressed after silencing GNG5 (Fig. 8B‒D; Fig. 9A‒C).

## Discussion

Despite advances regarding the treatment of cancer,^27^^,^^28^ the pathogenesis of CC remains poorly understood.^29^ In CC, circRNAs are implicated in many physiological and pathological processes.^30^^,^^31^ A novel circRNA, called circ-PAPPA, was found to be abnormally expressed in CC tissues. Its expression correlated with TNM stage, lymphatic metastasis, tumor size, and distant metastasis. Moreover, high expression levels of circ-PAPPA predicted poor prognosis of CC patients, suggesting that circ-PAPPA could be used as a new diagnostic and prognostic biomarker for CC. Furthermore, knocking down circ-PAPPA inhibited CC cell growth, glucose uptake, and lactate production, whereas overexpression of circ-PAPPA did the opposite, implying that inhibition of circ-PAPPA could have a therapeutic effect on CC by inhibiting aerobic glycolysis.

It is well known that cancer cells often exhibit metabolic reprogramming, i.e., aerobic glycolysis.^32^ That is, even under aerobic conditions, most cancer cells produce ATP by converting glucose to lactate.^33^ As a result of aerobic glycolysis, cancer cells bypass mitochondrial oxidative phosphorylation and are able to compete for glucose with normal cells.^34^ In addition, avoiding mitochondrial oxidative phosphorylation reduces the overproduction of reactive oxygen species, thereby protecting cancer cells.^35^ Aerobic glycolysis is regulated by multiple signaling factors, including circRNAs.^36^^,^^37^ This study found that circ-PAPPA was up-regulated in CC, and inhibiting circ-PAPPA reduced CC cell proliferation, migration, invasion and aerobic glycolysis and promoted apoptosis, whereas enhancing circ-PAPPA did the opposite. Multiple studies have shown that circRNAs often act as miRNA sponges to participate in the functional regulation of mRNAs.^38^, ^39^, ^40^ For instance, in prostate cancer, circ-PAPPA targets and negatively regulates miR-515–5p.^16^ Accordingly, it is speculated that circ-PAPPA functions as a specific miRNA sponge in CC, and miR-656–3p was selected as its target.

Abnormal expression of miRNA has been fully confirmed to be associated with the pathogenesis of CC.^41^ The authors found that miR-656–3p expression was reduced in CC, which is consistent with a previous study.^42^ miR-656–3p is aberrantly expressed in several cancers, such as hepatocellular carcinoma,^43^ lung cancer,^44^ and nasopharyngeal carcinoma.^25^ Restoration of miR-656–3p and depletion of miR-656–3p were found to restrict and stimulate CC cell growth and glycolysis, respectively, by extensive analyses, further confirming the miR-656–3p downstream mechanism of circ-PAPPA involvement in CC progression. Furthermore, it was also found that miR-656–3p directly targets GNG5.

GNG5 has been considered to be associated with different malignancies^45^ and is involved in cortical development,^46^ chondrocyte apoptosis,^47^ and embryonic development.^48^ GNG5 expression is elevated in islets exposed to high glucose and is associated with glucose metabolism.^49^ In this study, it was shown that GNG5 was highly expressed in CC, and down-regulating GNG5 reversed the effect of up-regulation of circ-PAPPA on CC cells, implicating that circ-PAPPA regulates CC aerobic glycolysis by targeting the miR-656–3p/GNG5 axis.

The experimental results reveal for the first time that circ-PAPPA enhances aerobic glycolysis by regulating the miR-656–3p/GNG5 axis to promote CC progression, providing a new CC treatment approach. However, the present research also has certain limitations, that is, only in vitro cell experiments were conducted and only the effect of circ-PAPPA on CC cells was explored. The authors hope that in future studies, the action of circ-PAPPA in CC can be further verified by animal experiments, and other downstream targets of circ-PAPPA can be further explored.

## Conclusion

In sum, circ-PAPPA is highly expressed in CC, and its expression level correlates with various clinical features and poor prognosis of CC patients. The present results suggest that circ-PAPPA enhances aerobic glycolysis by targeting GNG5 through miR-656–3p to promote CC progression, profiling that circ-PAPPA is a novel oncogene and a potential diagnostic biomarker for CC.

## Ethics statement

All procedures performed in this study involving human participants were in accordance with the ethical standards of the institutional and/or national research committee and with the 1964 Helsinki Declaration and its later amendments or comparable ethical standards. All subjects were approved by the Ethics Committee of Shenzhen Hospital of Guangzhou University of Chinese Medicine (Approval nº 2017GZ60931). Each subject participating in this study provided written informed consent.

## Authors’ contributions

Conceptualization Ai Yuan Cai and CiAi Lai, Data curation AiYuan Cai and HuiShi Ye, YuanHong Lin and JinYun Li Formal analysis, Funding acquisition AiYuan Cai, Investigation DongSheng Fang, Methodology ZhongBin Pan, Project administration ZhiWei Li, Resources GuangLiang Luo, Software YanFang Huang and CiAi Lai, Supervision AiYuan Cai, Validation YanFang Huang, Visualization ZhongBin Pan, Writing - original draft AiYuan Cai and CiAi, Writing - review \& editing DongSheng Fang, ZhongBin Pan and ZhiWei Li.

## Funding

Supported by Shenzhen Hospital (Futian) of Guangzhou University of Chinese Medicine Research Project (No.GZYSY2024013).

## Declaration of competing interest

The author(s) declared no potential conflicts of interest with respect to the research, authorship, and/or publication of this article
